# The diagnostic value of soluble urokinase-type plasminogen activator receptor (suPAR) for the discrimination of vertebral osteomyelitis and degenerative diseases of the spine

**DOI:** 10.1186/s13018-019-1420-6

**Published:** 2019-11-14

**Authors:** Jan Simon Scharrenberg, Ayla Yagdiran, Julia Brinkmann, Maik Brune, Jan Siewe, Norma Jung, Esther Mahabir

**Affiliations:** 1 Comparative Medicine, Center for Molecular Medicine, University of Cologne, Robert-Koch-Straße 21, 50931 Cologne, Germany; 2 Department of Orthopedic and Trauma Surgery, University of Cologne, Cologne, Germany; 3 Department of Medicine I and Clinical Chemistry, Heidelberg University Hospital, Heidelberg, Germany; 4 Faculty of Medicine, University Hospital, Cologne, Germany; 5 Department I of Internal Medicine, Division of Infectious Diseases, University of Cologne, Cologne, Germany

**Keywords:** Vertebral osteomyelitis, Soluble urokinase-type plasminogen activator receptor (suPAR), Diagnostics, Bacterial infection, Biomarker

## Abstract

**Background:**

There is still a challenge in discriminating between vertebral osteomyelitis and degenerative diseases of the spine. To this end, we determined the suitability of soluble urokinase-type plasminogen activator receptor (suPAR) and compared the diagnostic potential of suPAR to CRP.

**Methods:**

Patients underwent surgical stabilization of the lumbar and/or thoracic spine with removal of one or more affected intervertebral discs, as therapy for vertebral osteomyelitis (*n* = 16) or for erosive osteochondrosis (control group, *n* = 20). In this prospective study, we evaluated the suPAR and CRP levels before (pre-OP) and after surgery (post-OP) on days 3–5, 6–11, 40–56, and 63–142.

**Results:**

The suPAR levels in vertebral osteomyelitis patients were significantly higher than those from controls pre-OP, 3–5 days post-OP, and 6–11 days post-OP. Significantly higher CRP levels were observed in the vertebral osteomyelitis group than in the controls pre-OP and 6–11 days post-OP. Levels of suPAR and CRP correlated positively in all patients in the pre-OP period: *r* = 0.63 (95% CI: 0.37–0.79), *p* < 0.0001. The values for the area under the receiver operating characteristics curve (AUC) for pre-OP and the overall model post-OP were 0.88 (95% CI: 0.76–1.00) and 0.84 (95% CI: 0.71–0.97) for suPAR, 0.93 (95% CI: 0.85–1.00) and 0.77 (95% CI: 0.62–0.93) for CRP, and 0.98 (95% CI: 0.96–1.00) and 0.91 (95% CI: 0.82–1.00) for the combination of suPAR and CRP. The AUC for suPAR pre-OP revealed an optimum cut-off value, sensitivity, specificity, NPV, and PPV of 2.96 ng/mL, 0.69, 1.00, 0.80, and 1.00, respectively. For CRP, these values were 11.58 mg/L, 0.88, 0.90, 0.90, and 0.88, respectively.

**Conclusion:**

The present results show that CRP is more sensitive than suPAR whereas suPAR is more specific than CRP. Moreso, our study demonstrated that improvement in the diagnostic power for discrimination of vertebral osteomyelitis and degenerative diseases of the spine can be achieved by a combination of both suPAR and CRP.

**Trial registration:**

ClinicalTrials.gov, NCT02554227, posted Sept. 18, 2015, and updated Aug. 13, 2019

## Introduction

Vertebral osteomyelitis is a primary infection of the end-plates of the vertebral bodies with secondary infection of the adjacent intervertebral discs [1]. Concomitant abscesses are detected in about a third of the patients, potentially leading to neurological deficits at a rate of approximately 20% [2, 3]. The overall incidence rate of vertebral osteomyelitis increased from 0.5 cases per 100,000 person years 1978–1982 to 2.2 in 1995 and 5.8 in 2008. It is most common among older persons with a higher incidence among men [3–5]. Clinical symptoms, especially in the early stages, are unspecific. Patients suffer from back pain, and fever occurs only in 50% of all cases [6]. Given that current markers including leucocyte count, erythrocyte sedimentation rate (ESR) and C-reactive protein (CRP) are also unspecific, several weeks may elapse between the first symptoms and the final diagnosis of vertebral osteomyelitis [3, 7].

Vertebral osteomyelitis is primarily caused by hematogenous seeding leading to monomicrobial infections. *Staphylococcus aureus* is most frequently isolated followed by streptococci species and *Escherichia coli* [2, 8] while coagulase-negative staphylococci are more often found after spinal surgery [9]. Nevertheless, worldwide vertebral osteomyelitis is mostly caused by *Mycobacterium tuberculosis,* and brucellosis is more frequently found than pyogenic infection in the Mediterranean and Middle East countries [10].

To identify the pathogens for an effective therapy tailored to the causative agent, blood cultures, computed tomography (CT)-guided fine-needle aspiration or open biopsies [11, 12] may be needed. Nevertheless, also due to previous antibiotic treatment the pathogen can only be identified in approximately two thirds of the patients [5, 13]. Magnetic resonance imaging (MRI) is the gold standard of imaging to detect vertebral osteomyelitis [14].

Treatment of an advanced vertebral osteomyelitis consists of removal of the necrotic tissue, stabilization of the affected vertebral bodies and concomitant antibiotic therapy [15]. Currently, there are different recommendations for the duration of antibiotic treatments but 6 weeks were shown to be suitable [16]. For evaluating the therapy response, clinical improvement and the CRP value are used. Nevertheless, due to the low specificity of CRP, new biomarkers are needed for improvement of diagnosis and treatment monitoring to prevent long periods with symptoms and destructive changes of the spine.

The urokinase plasminogen activator (uPA) is a proteolytic enzyme, which converts the proenzyme plasminogen to the active serine protease plasmin [17]. The urokinase-type plasminogen activator receptor (uPAR) is a glycoprotein, which is expressed on various immunologically active cells, and is released during inflammation and infection. uPAR is cleaved from the cell surface by proteolysis to produce the soluble urokinase-type plasminogen activator receptor (suPAR), which can be found in urine, blood, and cerebrospinal fluid [18]. The suPAR levels are low in healthy patients [17, 19] while levels are significantly increased during immune activation [20, 21]. A recent report showed that suPAR correlated highly with the C-reactive protein (CRP) in patients with prosthetic joint infection [21].

Our goal was to establish a non-invasive method, which allows discrimination of vertebral osteomyelitis and degenerative diseases of the spine. The potential of such a diagnostic method lies in the reduction of morbidity and mortality due to vertebral osteomyelitis and reducing medical costs. To this end, blood samples from patients with vertebral osteomyelitis or erosive osteochondrosis (a non-infectious, degenerative disease of the spine with similar surgical treatment as vertebral osteomyelitis) were collected and analyzed for suPAR levels.

## Materials and methods

### Study participants

The present study is a prospective single-center case-control study. The patients included were recruited in the Department of Orthopedic and Trauma Surgery of the University Hospital of Cologne. In all cases of vertebral osteomyelitis, the diagnosis was confirmed by clinical (back or leg pain), microbiological, and imaging (MRI or CT if MRI was contraindicated, as with Patient 2) results. Detection of a virulent organism such as *Staphylococcus aureus* and Gram-negative bacteria in at least one relevant sample or the detection of a low-virulent organism such as coagulase-negative staphylococci or *Propionibacterium spp.* in at least two relevant samples was considered as the etiologic pathogen. The patients underwent surgical stabilization of the lumbar and/or thoracic spine in combination with removal of one or more affected intervertebral discs, either as therapy for vertebral osteomyelitis (*n* = 16; 10 males, 6 females) (Table 1) or for erosive osteochondrosis (control group, *n* = 20; 9 males, 11 females) (Table 2).

**Table 1:** Demographic and past or current clinical features of the vertebral osteomyelitis patients

Patient	Age (years)	Gender (m/f)	Secondary diagnoses	Days of blood draw				
				Pre-OP	3–5 days post-OP	6–11 days post-OP	40–56 days post-OP	63–142 days post-OP
1	76	m	NPP with surgery L2/L3 left side	0	5	9	n.a.	98
2	79	m	CHD, ischemic cardiomyopathy with low left-ventricular function, implantation of defibrillator, chronic sigma diverticulitis, partly gastric resection, glomus tumor	0	5	9	n.a.	96
3	58	f	AH, CMV infection, paroxysmal atrial fibrillation, obstructive sleep apnea syndrome, hypothyreosis, obesity, hepatic steatosis, renal insufficiency, hyperbilirubinaemia (Morbus Meulengracht), cholecystectomy, hysterectomy	-1	4	8	48	104
4	66	m	AH, type 2 DM, diabetic foot syndrome, diabetic nephropathy, atrial septal aneurysm, hepatitis E infection, type C-gastritis, middle-grade valvular aortic stenosis, mitral insufficiency grade 1, obstructive sleep apnea syndrome, borreliosis	-1	3	9	50	94
5	71	m	AH, urosepsis, acute renal failure with initial creatinine of 2.2 mg/dl, paroxysmal atrial fibrillation, middle-grade aortic stenosis and low-grade insufficiency with high calcification of the aortic valve, hypothyroidism, incomplete disc herniation in thoracic/lumbal spine	-1	5	11	43	85
6	53	f	None	-1	3	11	50	99
7	68	f	AH, osteomyelitis in childhood, gastric ulcer	-14	3	9	43	85
8	75	f	AH, urosepsis, thrombophlebitis, CHD, total knee arthroplasty right leg	0	5	9	56	126
9	63	m	Bradycardia, pacemaker	0	5	9	42	83
10	71	m	AH, hepatitis A infection, hip total endoprosthesis 3× left side caused by empyema, shoulder surgery left side caused by empyema	0	3	7	47	103
11	54	m	Deep vein thrombosis right leg, fracture of the left femur and left lower leg	0	3	11	41	90
12	72	f	AH, ovarial cancer, urethral splint, transient ischemic attack	0	3	9	47	98
13	59	m	AH, deep vein thrombosis	-8	4	8	40	110
14	77	m	AH, cholecystectomy, benign prostate hyperplasia, aneurysm rupture with hemiparesis accented right arm	-9	n.a.	8	41	123
15	85	m	AH, atrial fibrillation, decompression of lumbal spine	-20	n.a.	9	n.a.	66
16	73	f	AH, DM, dorsal spondylodesis	-1	3	10	n.a.	63

*m* male, *f* female, *L* lumbar, *NPP* nucleus pulposus prolapse, *CHD* coronary heart disease, *AH* arterial hypertension, *CMV* cytomegalovirus, *DM* diabetes mellitus, *pre-OP* before surgery, *post-OP* after surgery, *day of surgery* day 0, *n.a.* complete blood draw missing

**Table 2:** Demographic and past or current clinical features of the control patients

Patient	Age (years)	Gender (m/f)	Secondary diagnoses	Days of blood draw				
				Pre-OP	3–5 days post-OP	6–11 days post-OP	40–56 days post-OP	63–142 days post-OP
21	80	f	AH, DM, hypothyreosis, CHD, inner ear hearing loss left side	0	4	8	42	96
22	75	f	AH, 3× decompression of lumbal spine	-1	3	8	42	91
23	70	f	AH, CHD, metabolic syndrome, obesity	-1	4	10	40	96
24	74	f	Attack of gout	0	3	7	48	90
25	78	f	AH, colonic carcinoma	-1	5	7	42	91
26	54	m	Nucleotomy L4/L5, decompression L5/S1, CHD with coronary by-pass surgery, PAD stented *A. ilica communis*	0	4	8	41	90
27	57	f	AH, facet joint cyst removal + foraminotomy L5 left	0	3	9	40	91
28	58	m	AH, atrial fibrillation	0	4	7	41	83
29	66	m	AH, gastro-esophageal reflux disease, vertebral instability L4/5, stenosis of neuroforamina L4 right side, decompression surgery of spinal stenosis L4/5 left side	0	n.a.	6	48	n.a.
30	63	f	AH, acute renal, PAD multiple femoropopliteal by-pass surgery in both legs, occluded by-pass right leg, de novo scoliosis L3-L5 with absolute spinal stenosis	0	n.a.	9	43	142
31	52	m	None	0	3	6	43	92
32	63	f	AH, hypothyreosis	0	4	n.a.	44	91
33	72	m	AH, Spondylolisthesis	-1	3	7	40	89
34	59	f	*sigma diverticulitis*, gastritis, esophageal varices, nodular goiter, alcohol-toxic liver cirrhosis, type 2 DM	-1	n.a.	7	48	104
35	77	m	AH, DM type 2, CHD, dual coronary by-pass surgery, PAD, by-pass surgery in both legs, lumbar fusion surgery L3-L5 with screw burst L5	0	3	9	44	86
36	72	f	osteoporosis, old compression fracture Th11, Th12, L3, incomplete fracture Th10	0	4	9	41	86
37	60	m	None	0	n.a.	7	43	92
38	72	f	AH, DM type 2, dyslipoproteinaemia, breast cancer, vitamin D deficiency	0	n.a.	7	42	91
39	53	m	AH	-1	n.a.	11	41	90
40	61	m	AH, aortic valve stenosis, dyslipoproteinaemia, hypothyreosis, CHD	0	3	8	n.a.	86

*m* male, *f* female, *L* lumbar, *S* sacral, *Th* thoracic, *AH* arterial hypertension, *DM* diabetes mellitus, *CHD* coronary heart disease, *PAD* peripheral artery disease, *pre-OP* before surgery, *post-OP* after surgery, *day of surgery* day 0, *n.a.* complete blood draw missing

The eligibility criteria for the control and vertebral osteomyelitis groups were an age between 40 and 85 years, both sexes, lumbar spine pathology with an indication of vertebral osteomyelitis or erosive osteochondrosis and a medical indication of surgical stabilization of affected lumbar and/or thoracic vertebral bodies, full legal competence, and the existence of a written informed consent. The exclusion criteria were the existence of autoimmune diseases, acute or chronic infections such as human immunodeficiency virus (HIV), hepatitis B or C, acute infections of other parts of the body besides the spine, and cancer.

For surgery, all patients received intravenous general anesthesia in combination with intubation. Additionally, all control patients received perioperative antibiotic treatment with 2 g of cefazolin. To identify the causative pathogen, blood cultures were taken prior to and during surgery. Also, tissue samples were obtained during surgery for microbiological analysis. The causative pathogen was identified by reviewing all microbiological results by an experienced infectious disease specialist (NJ). The diagnosis of vertebral osteomyelitis was confirmed by evaluation of microbiological, clinical, and imaging findings by NJ and AY (Table 3).

**Table 3:** Clinical features of the vertebral osteomyelitis patients, as determined by microbiological analysis of blood cultures or biopsies

Patient	Infectious agent	Microbiological method	Imaging method
1	*S. epidermidis*	5× biopsy	MRI
2	*S. epidermidis*	2× biopsy	CT
3	*S. aureus* (MRSA)	3× biopsy	MRI
4	*Streptococcus dysgalactiae*	1× blood culture	MRI
5	*E. coli*	4× blood culture	MRI
6	*S. epidermidis*	3× biopsy, 1× blood culture	MRI
7	*E. coli*	2× biopsy	MRI
8	*S. aureus* (MSSA)	2× biopsy	MRI
9	*S. epidermidis*	2× biopsy	MRI
10	*Parvimonas micra*	3× biopsy	MRI
11	*Proprionibacterium acnes*	3× biopsy	MRI
12	*Streptococcus dysgalactiae*	5× biopsy, 1× blood culture	MRI
13	*S. aureus* (MSSA)	3× biopsy, 1× blood culture	MRI
14	*S. aureus* (MSSA), *E. coli*	*S. aureus* (MSSA): 2× biopsy, 2× blood culture; *E. coli:* 4× biopsy, 4× blood culture	MRI
15	*E. coli*	3× biopsy	MRI
16	*S. lugdunensis*	2× biopsy	MRI

*S.* Staphylococcus, *MRSA* methicillin-resistant *S. aureus*, *MSSA* methicillin-sensitive *S. aureus*, *E. coli Escherichia coli*. *MRI* magnetic resonance imaging, *CT* computed tomography

All relevant data of the patients were documented, including age, sex, body mass index (BMI), nicotine and alcohol abuse, medication, co-morbidities, clinical symptoms, diagnostic procedures and results, type of surgery, implant material used, and medical complications. The demographical data and clinical features of the patients are shown in Tables 1 and 2.

### Blood draws and serum preparation for suPAR measurements

Blood samples were taken at five defined timepoints from each patient (Table 1 and Table 2), before surgery (pre-OP) and after surgery (post-OP): 3–5 days, 6–11 days, 40–56 days, and 63–142 days. Due to other medical treatments, it was not possible to take blood samples 3–5 days post-OP from 2 patients (14, 15), 40–56 days post-OP from 5 patients (1, 2, 15, 16, 40), and 63–142 days post-OP from one patient (29). Only values from timepoints with both a valid suPAR measurement and a corresponding valid CRP level were included in the statistical analysis. For clarity, the group sizes are shown in Table 4.

**Table 4:** No. of patients from the different intervals that were included in the statistical analysis

Interval	Group size (no. of patients)	
	Spondylodiscitis	Controls
Pre-OP	16	20
3–5 days post-OP	14	14
6–11 days post-OP	16	19
40–56 days post-OP	12	19
63–142 days post-OP	16	19
Overall post-OP	11	12

After an overnight fast and while the patient was in a lying position, blood draws from peripheral veins of the lower arm or the back of the hand or from a central venous catheter were performed at the Department of Orthopedic and Trauma Surgery, University Hospital of Cologne. In cases of puncture of peripheral veins, the stasis was maintained for a maximum time of 2 min.

Blood for the suPAR measurements was collected in serum gel tubes (S-Monovette® Serum-Gel 4.7 mL, Sarstedt, Nümbrecht, Germany). The samples were kept for 30 to 45 min in an upright position to allow coagulation and then centrifuged at 3461×*g* for 5 min (EBA 20 Centrifuge, Hettich Lab Technology, Tuttlingen, Germany). The serum was then aliquoted and stored in storage tubes (NuncTM CryoTubeTM 1.8 mL, ThermoFisher Scientific, Waltham, USA) at − 80 °C until analysis.

### CRP level determination

For determination of CRP, blood was drawn as described above in lithium-heparin tubes (S-Monovette®, lithium-heparin, Sarstedt). It was centrifuged at 4000 g and 21 °C for 10 min. Plasma was aliquoted within 3 h after blood drawing and used fresh. The CRP level was determined via latex agglutination assay according to the manufacturer’s instructions (C-Reactive Protein Gen.3, cobas®, Roche Diagnostics, Basel, Switzerland). Briefly, plasma was diluted 1:100 and added on a slide, which was pre-coated with antibodies to monoclonal anti-human CRP and latex reagent. After 2 min incubation, clear agglutination was observed on the slide and it was examined turbidimetrically using the analytic system cobas® C702 (Roche Diagnostics). CRP values below 3 mg/L are considered clinically irrelevant and were adjusted to 0 mg/L. Values ≥ 5 mg/L were classified as pathological.

### suPAR measurements

For this study, the Human uPAR Quantikine® ELISA kit (R&D Systems, Minneapolis, USA) was used according to the manufacturer’s instructions. The measurement of the optical densities was performed by the use of the “Infinite 200 Pro” plate reader (Tecan Group Ltd., Männedorf, Switzerland). In this study, all suPAR measurements were performed in duplicate. The suPAR concentrations were determined by calculating the average optical density value of the two wells with the same sample and determining the suPAR value by the use of the interpolated standard curve.

### Statistical analyses

The values for suPAR (ng/mL) and CRP (mg/L) are provided as mean ± standard error of the mean (SEM). Differences between the vertebral osteomyelitis and the control groups at the same intervals were assessed by two-sample *t* tests allowing for heterogeneity of the variances (method Satterthwaite). The correlations between suPAR and CRP (pre-OP and post-OP overall) and stratified by the sampling intervals and by sex were estimated and tested employing the Spearman rank correlation co-efficient. Overall post-OP and period-specific logistic regression models were set up for determining the detection of vertebral osteomyelitis based on the biomarkers suPAR and CPR. The logistic regression models were adjusted accordingly for sex and the corresponding sex*biomarker interactions. The Wald-chi-square statistic served to assess the significance of the effects (*p* values) in the logistic regression models. To prevent confounding the mean values for overall post-OP, only data from patients with available suPAR and CRP values at all 4 timepoints were used for overall post-OP calculations (vertebral osteomyelitis group: *n* = 11, controls: *n* = 12), Table 4.

To investigate the predictive quality of different alternative models, receiver operating characteristic (ROC) curves were considered. A guide for classifying the accuracy of a diagnostic test based on AUC (area under the curve) values is 0.91–1.00: excellent, 0.81–0.90: good, 0.71–0.80: fair, 0.61–0.70: poor, and 0.51–0.60: fail. The sensitivity and specificity as well as positive and negative predictive values for suPAR and CRP were computed together with their 95% confidence intervals for the cut-off level. The Youden’s index with the highest sum of the sensitivity and specificity was used to select the optimal cut-off for analysis.

Differences or effects estimates with *p* values < 0.05 were considered statistically significant. For statistical analyses, we used GraphPad Prism 7 (La Jolla, CA, USA), R 3.2.1, Wolfram MATHEMATICA 11.3) and mostly SAS/STAT software UE (SAS Institute Inc.: SAS/STAT User’s Guide, Cary NC: SAS Institute Inc., 2014).

### Ethics

This study was performed according to the Helsinki guidelines in compliance with national regulations for the use of human material. Utilization of human blood samples and tissues for research purposes was approved by the Ethics Committee of the University of Cologne (reference number: Uni-Köln 9-2014). This study is registered with a ClinicalTrials.gov identifier number of NCT02554227. All patients gave written informed consent before participation in this study.

## Results

To determine the suitability of suPAR for vertebral osteomyelitis diagnosis, the suPAR concentrations in serum from vertebral osteomyelitis patients (*n* = 16) and from a control group with erosive osteochondrosis (*n* = 20) were measured pre-OP and post-OP (3–5 days, 6–11 days, 40–56 days, and 63–142 days). The suPAR and CRP concentrations were compared at each interval within each group.

**Fig. 1:**
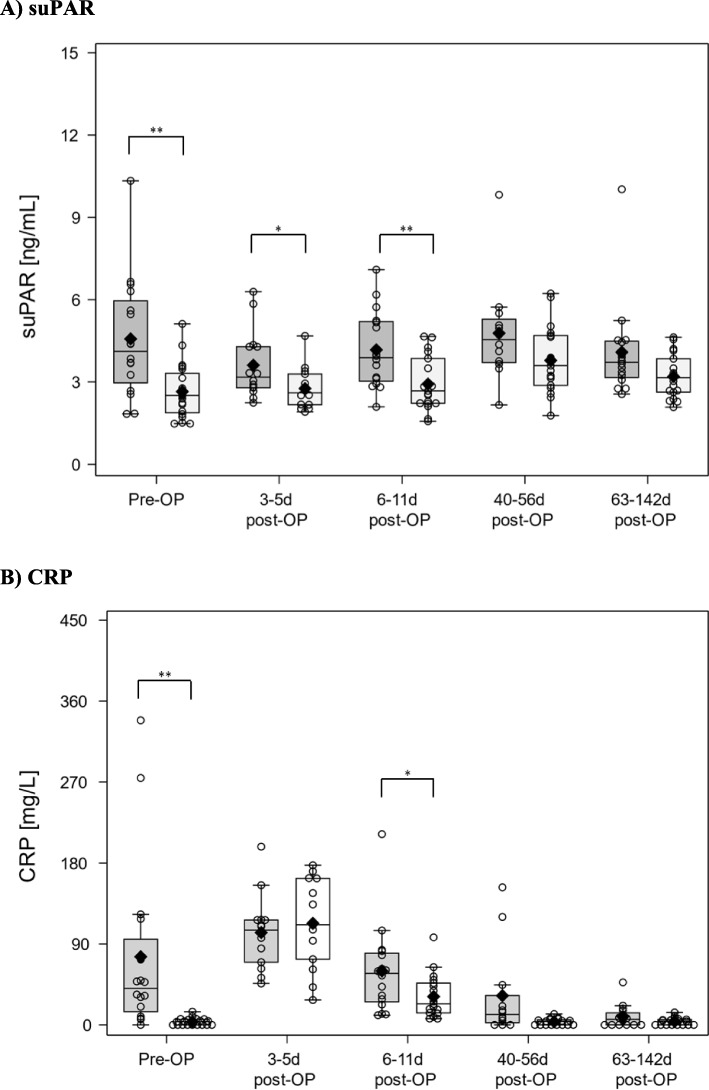
suPAR serum levels (**a**) and CRP plasma levels (**b**) in vertebral osteomyelitis (dark gray bars) and control patients (light gray bars). Pre-OP, before surgery; post-OP, after surgery. The absolute suPAR concentration (ng/mL) and CRP concentration (mg/L) are indicated on the *Y*-axis. Box-and-whiskers plot; data points, open circles; maximum, endpoint of upper whisker; minimum, endpoint of lower whisker; third quartile (75th percentile), upper edge of the box; first quartile (25th percentile), lower edge of the box; median (50th percentile), line inside the box; mean, black diamond; data points beyond the whiskers, outliers. Results are expressed as mean ± SEM. Significant differences in concentrations are marked as follows: *p < 0.05, **p < 0.01

Due to variations of more than 20% between the duplicate measurements for suPAR, 6 values were excluded 3-5 days post-OP from control patients 29, 30, 34, 37, 38, and 39 and because of the lack of a valid corresponding CRP value, the 6–11 days post-OP value of control patient 32 was excluded. Mean values of suPAR concentrations ranged from 3.61 ± 0.33 (3–5 days post-OP) to 4.78 ± 0.54 ng/mL (40–56 days post-OP) in vertebral osteomyelitis patients while these values were 2.65 ± 0.22 (pre-OP) to 3.79 ± 0.28 ng/mL (40–56 days post-OP) in controls (Fig. 1a). Generally, within the same interval, suPAR values from vertebral osteomyelitis patients were higher than those from controls (pre-OP, *p* = 0.0041; 3–5 days post-OP, *p* = 0.0402; 6–11 days post-OP, *p* = 0.0060; 40–56 days post-OP, *p* = 0.1192; 63–142 days post-OP, *p* = 0.0744) and were, therefore, significantly different from each other pre-OP, 3–5 days post-OP, and 6–11 days post-OP. Over all post-OP intervals, differences between the vertebral osteomyelitis group and the controls were significant (*p* = 0.0167).

The CRP values for both patient groups are shown in Fig. 1b. In the vertebral osteomyelitis patients, the CRP concentration was 75.75 ± 24.44 mg/L pre-OP and increased to 102.73 ± 10.84 mg/L 3–5 days post-OP, decreasing continuously until the end of the study to 9.29 ± 3.08 mg/L. A similar pattern was observed for the controls. Concentrations increased from 3.49 ± 0.90 mg/L pre-OP to 112.99 ± 13.06 mg/L 3–5 days post-OP and decreased to 3.45 ± 0.82 mg/L 40–56 days post-OP and 3.8 ± 0.91 mg/L 63–142 days post-OP. Significantly higher CRP values were observed in the vertebral osteomyelitis group than in the controls pre-OP (*p* = 0.0098) and 6–11 days post-OP (*p* = 0.048). Over all post-OP intervals, differences between the vertebral osteomyelitis group and the controls were significant (*p* = 0.0490).

Measurements for suPAR and CRP were positively correlated in the vertebral osteomyelitis group in the pre-OP period, *r* = 0.55 (95% CI: 0.07–0.82), *p* = 0.023, and in all patients in the pre-OP period, *r* = 0.63 (95% CI: 0.37–0.79), *p* < 0.001 and 6–11 days post-OP, *r* = 0.45 (95% CI: 0.13–0.68), *p* = 0.0059. However, for the overall post-OP period, the suPAR and CRP correlation was positive but not significant; *r* = 0.39 (95% CI: − 0.04 to 0.68), *p* = 0.0688. In the controls pre-OP as well as overall post-OP, the suPAR and CRP correlations were not significant, pre-OP: *r* = − 0.06 (95% CI: − 0.49–0.40), *p* = 0.8082 and post-OP: *r* = 0.14 (95% CI: − 0.48 to 0.66), *p* = 0.6706.

**Fig. 2:**
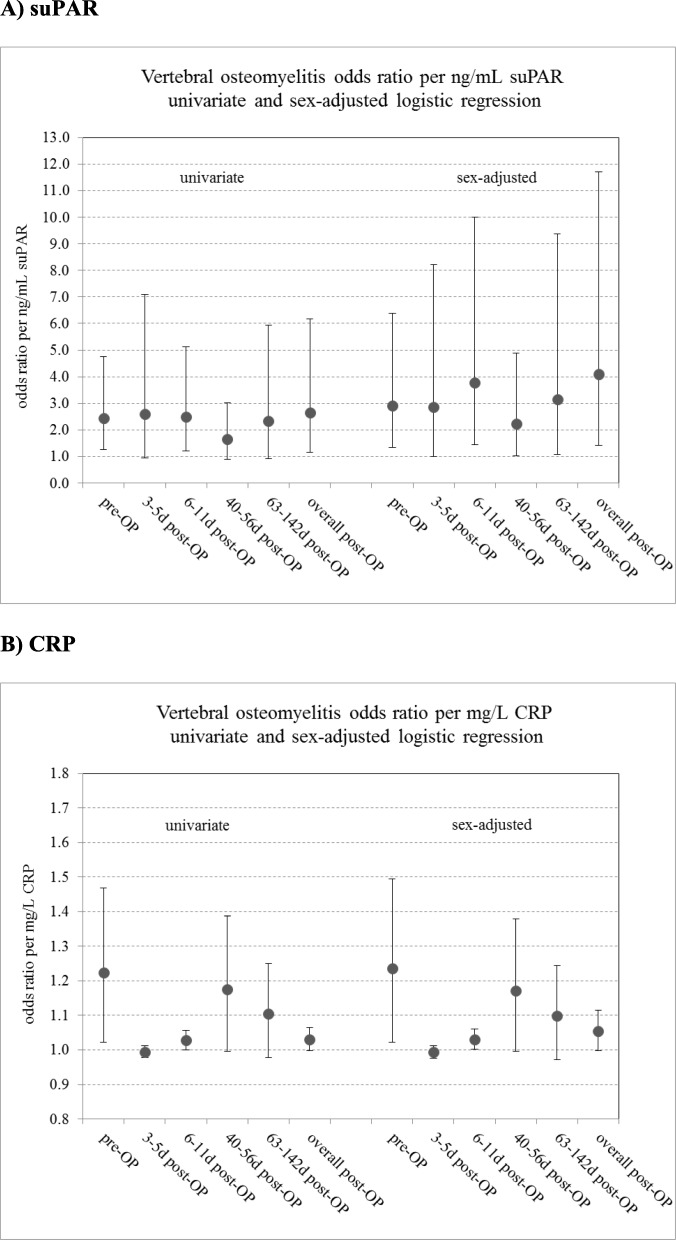
Odds ratios and 95% CI of the univariate and sex-adjusted logistic regression of patient status (vertebral osteomyelitis vs. control patients) with respect to suPAR (**a**) and CRP (**b**) each stratified by the interval pre-OP, post-OP, and all post-OP intervals combined (overall post-OP)

Figure 2 summarizes the main findings of the logistic regression analyses for suPAR and CRP stratified by interval and overall post-OP for all intervals post-OP. Figure 2a and Fig. 2b show the odds ratios together with their 95% CI by interval and overall post-OP for suPAR and CRP, respectively. Logistic regression of patient status with respect to suPAR as well as CRP and adjusted for sex reveals a significant predictive potential of these parameters for diagnosis of vertebral osteomyelitis for both pre-OP as well as for post-OP overall. For example, the odds ratio in the univariate logistic regression for suPAR pre-OP is 2.46 (95% CI: 1.27–4.76), *p* = 0.0078. This means that the odds of developing vertebral osteomyelitis increases by the factor 2.46 per change in the suPAR measurement by 1 ng/mL. Adjusting for the sex of the patients increases the odds ratio per ng/mL to 2.92 (95% CI: 1.34–6.38), *p* = 0.0071. Likewise, the odds ratio in the univariate logistic regression for CRP pre-OP is 1.22 (95% CI: 1.02–1.47), *p* = 0.0278. This means that the odds of developing vertebral osteomyelitis increases by the factor 1.22 (i.e., 22% increase) per change in the CRP measurement by 1 mg/L. Adjusting for the sex of the patients increased the odds ratio slightly to 1.24 (95% CI: 1.02–1.5), *p* = 0.0281.

**Fig. 3:**
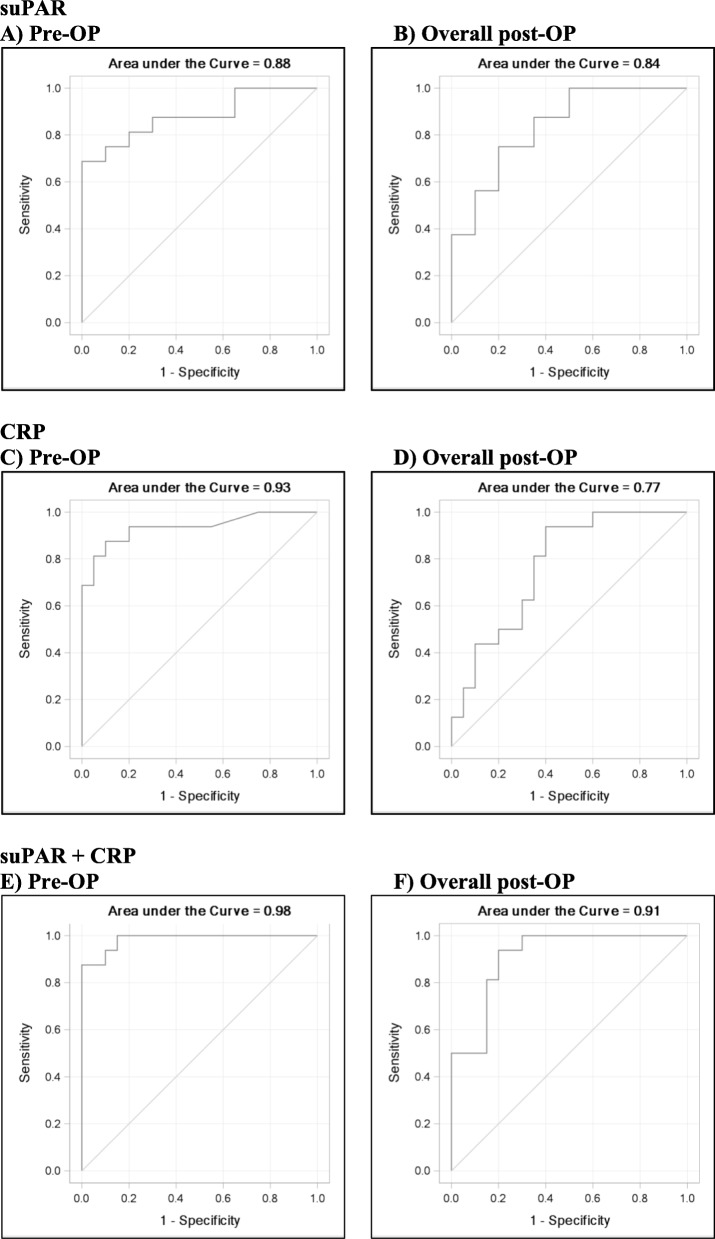
ROC curves for sex-adjusted logistic regression analyses of patient status with respect to suPAR, CRP, and the combination of suPAR and CRP; logistic models stratified by the interval pre-OP and all post-OP intervals combined (overall post-OP); AUC and 95% CI for pre-OP and for overall post-OP are 0.88 (95% CI: 0.76–1.00) and 0.84 (95% CI: 0.71–0.97) for suPAR (**a**, **b**), 0.93 (95% CI: 0.85–1.00) and 0.77 (95% CI: 0.62–0.93) for CRP (**c**, **d**), and 0.98 (95% CI: 0.96–1.00) and 0.91 (95% CI: 0.82–1.00) for the combination of suPAR and CRP (**e**, **f**), respectively

The accuracy of a diagnostic test depends on how well the test separates the group being tested into those with and without the disease or condition in question. Receiver operating characteristics (ROC) curve analysis revealed that the values for the AUC based on logistic regression of patient status with respect to suPAR and CRP measurements and adjusted for sex were 0.88 (95% CI: 0.76–1.00) and 0.93 (95% CI: 0.85–1.00) for pre-OP, and 0.84 (95% CI: 0.71–0.97) and 0.77 (95% CI: 0.62–0.93) for the overall model post-OP, respectively, as shown in Fig. 3 and Table 5. The AUC based on logistic regression for the combination of suPAR and CRP and likewise adjusted for sex showed higher results both in the pre-OP, 0.98 (95% CI: 0.96–1.00), as well as in the overall post-OP period, 0.91 (95% CI: 0.82–1.00). The cut-off levels, sensitivity, specificity, positive predictive value (PPV), and negative predictive value (NPV) for suPAR and CRP for diagnosis of vertebral osteomyelitis are shown in Table 5.

**Table 5:** Diagnostic value of serum levels of suPAR and CRP for distinguishing between vertebral osteomyelitis and degenerative diseases of the spine

Parameter	Interval	AUC	95% confidence interval	Cut-off	Sensitivity	Specificity	Odds ratio	Positive predictive value	Negative predictive value	Youden’s index
suPAR (ng/mL)	Pre-OP	0.88	0.76–1.00	2.960	0.688	1.000	-*	1.000	0.800	0.688
	Overall post-OP	0.84	0.71–0.97	4.021	0.750	0.800	12.00	0.750	0.800	0.550
CRP (mg/L)	Pre-OP	0.93	0.85–1.00	11.580	0.875	0.900	63.00	0.875	0.900	0.775
	Overall post-OP	0.77	0.62–0.93	63.210	0.938	0.600	22.50	0.652	0.923	0.538

*suPAR* soluble urokinase-type plasminogen activator receptor, *CRP* C-reactive protein, *pre-OP* before surgery, *post-OP* after surgery, *AUC* the area under the receiver operating characteristics curve adjusted for sex

*No value due to a 0 in the denominator

The optimal cut-off values were determined using ROC curve analysis and Youden’s index

## Discussion

Current diagnostic methods for vertebral osteomyelitis are based on structural changes in the spine, delaying early diagnosis and treatment. To the best of our knowledge, this is the first study to explore the potential of suPAR for differentiating between vertebral osteomyelitis and degenerative diseases of the spine. Microbiological analyses are necessary to identify the causative pathogen. Notably, the current results show that suPAR is a suitable adjunct biomarker to CRP for diagnosing vertebral osteomyelitis. Furthermore, the potential for diagnosing vertebral osteomyelitis before surgery was higher with CRP than with suPAR, the latter showing a higher specificity. The diagnostic potential of the combination of both biomarkers was superior to the use of the single biomarkers prior to surgery as well as in the post-OP period.

To date, there is only one report about suPAR concentrations and diseases of the spine [22]. Toldi et al. found plasma suPAR levels of 2.57 to 3.80 ng/mL in patients suffering from ankylosing spondylitis and 2.06 to 3.42 ng/mL in healthy patients, therefore showing no significant differences between both groups. In contrast, in the present study, suPAR values were significantly higher in vertebral osteomyelitis patients ranging from 3.61 to 4.78 ng/mL compared to 2.65 to 3.79 ng/mL in controls. The differences in the results obtained by Toldi et al. and our results for vertebral osteomyelitis could be because ankylosing spondylitis is an immune-mediated rheumatoid disease resulting in chronic inflammation in the vertebrae with systemic manifestations at a later stage of this mild disease [22].

In the present study, a significant positive correlation between suPAR and CRP was found only prior to surgery in the vertebral osteomyelitis group. A positive correlation between suPAR and CRP was also reported for critically ill intensive care patients with or without sepsis [23] and prosthetic joint infection [21]. However, none was found in patients with rheumatic diseases [24], pneumococcal bacteraemia [25], and severe sepsis [26]. Therefore, the mostly weak and insignificant correlations between suPAR and CRP in the post-OP intervals in the vertebral osteomyelitis group in the current study are consistent with the latter reports.

In the plasma of healthy humans, suPAR is found in low constant concentrations [17, 19]. Increased suPAR levels were found in several bacterial diseases including bacteraemia [25, 27–30], sepsis [26, 31], tuberculosis [31], purulent meningitis [32], and prosthetic joint infections [21]. Previous reports show that suPAR levels approximated 1.0 to 20.0 ng/mL in patients with different infections [21, 22, 33, 34]. The suPAR levels were summarized by Eugen-Olsen [20] to be < 4 ng/mL in healthy, > 4 < 10 ng/mL for low-grade inflammation, and > 10 ng/mL for critical illness. The suPAR levels determined in the present study averaged 3.61 to 4.78 ng/mL in the vertebral osteomyelitis patients while concentrations in the control group were 2.65 to 3.79 ng/mL. According to the classification of Eugen-Olsen for suPAR, vertebral osteomyelitis in our patient cohort can be considered a low-grade infection.

Cut-off levels for suPAR may be used for diagnosis but this approach would depend on the patient cohort and disease of concern. Cut-off levels, sensitivities, and specificities, respectively, were reported to be 10.0 ng/mL, 0.38, and 0.95 for diagnosis of *Streptococcus pneumoniae* bacteraemia [25], 2.7 ng/mL, 0.35, and 0.67 for diagnosis of bacterial infection in SIRS patients [35], and 2.96 ng/mL, 0.69 and 1.00 in the present study. Therefore, suPAR measurement may be useful in monitoring the therapy response in patients. After 8 months of treatment for tuberculosis in patients with and without HIV, suPAR levels decreased significantly by 0.56 to 2.07 ng/mL among sputum-positive patients, levels being comparable to those of tuberculosis-negative patients [31]. Ostrowski et al. also reported a decrease in plasma suPAR 1 year after the induction of therapy in patients suffering from HIV who had a high baseline suPAR level [36]. Significant decreases in suPAR levels were also observed subsequent to a 4-to-7 day antimicrobial therapy for SIRS in children [37]. In the present study, the vertebral osteomyelitis patients received antibiotics perioperatively and post-OP. In contrast to the CRP levels, which decreased with time, no significant decrease was observed in the suPAR levels in both groups throughout the study. To the authors’ knowledge, there are no reports concerning the mechanism responsible for this observation. Since two of the abovementioned studies also revealed decreasing suPAR values within longer periods of follow-up, notably 8 months to 1 year [31, 36], it is possible that the duration of the present study of up to 5 months was insufficient to observe decreasing suPAR concentrations. Therefore, the present data show that monitoring of the therapy success can be performed using CRP but not suPAR.

Specific inflammation parameters are needed in the diagnostic work-up and evaluation of treatment success of vertebral osteomyelitis, especially in cases with low-virulent causative agents where CRP values are normal or low. A greater challenge is posed because the CRP value alone is not always helpful to distinguish between vertebral osteomyelitis and degenerative diseases of the spine. As shown in the present study, suPAR is only elevated in vertebral osteomyelitis patients and therefore is a specific biomarker for differentiating between vertebral osteomyelitis and degenerative diseases of the spine pre-operatively. Therefore, in difficult cases, additional specific parameters such as suPAR are needed to determine the pre- and intra-operative diagnostic pathways. Notably, the significantly different concentrations of suPAR in the patients with vertebral osteomyelitis compared to the control patients shortly after surgery could reveal a potential use of suPAR in diagnosing the infection since CRP is of limited use for this purpose also due to the strong influence of surgery on the non-specific CRP concentration [38, 39].

There are many strengths of the present study. We were able to do a 5-month follow-up with 5 intervals in patients, thus increasing the impact of the study. The suPAR assay employed in the present study is a double monoclonal antibody sandwich assay, which measures all circulating suPAR including full-length and cleaved forms of the receptor. Furthermore, suPAR is highly stable in serum and plasma for 24 h at room temperature [40, 41] or 72 h at 4 °C [40] and is not affected by circadian rhythm [42], repeated freeze-thaw cycles [40, 41] nor surgery [43, 44]. The latter results were also confirmed in the present study for suPAR, in contrast to CRP, where CRP values were comparable for both groups 3-5 days post-OP.

However, there are some limitations of this study. Due to the fact that suPAR levels are also elevated due to co-morbidities, some of which have been mentioned above, it is considered a non-specific biomarker. Since suPAR concentrations may remain stable for a long period after treatment, as mentioned above, it may not be a suitable marker for monitoring the therapy success. Even though the patients’ co-morbidities were reported consistently, the influence of possible undetected diseases on suPAR levels cannot be excluded which may have an impact on the mean values because of the relatively small number of patients included in this single-center study. Furthermore, there is a certain form of erosive osteochondrosis (MODIC Type 1), which has an immunological active character [45, 46]. As it is not investigated yet, the effect of this form on the suPAR concentrations remains unclear and should be part of further studies. Therefore, control patients should be examined by imaging as was done with the vertebral osteomyelitis group.

## Conclusions

Our results show that suPAR is more specific than CRP whereas CRP is more sensitive than suPAR for discrimination of vertebral osteomyelitis and degenerative diseases of the spine. Furthermore, improvement in the diagnostic potential can be achieved by a combination of both suPAR and CRP. Also, the present study reveals a potential use of suPAR as a biomarker for detection of post-operative infections and therefore, opportunities for further research.

## Data Availability

The datasets used and/or analyzed during the current study are available from the corresponding author on reasonable request.

## Abbreviations

CRP: C-reactive protein
suPAR: Soluble urokinase-type plasminogen activator receptor
